# Year-round at-sea movements of fairy prions from southeastern Australia

**DOI:** 10.1098/rsos.220134

**Published:** 2022-05-24

**Authors:** Aymeric Fromant, Yonina H. Eizenberg, Timothée Poupart, Paco Bustamante, John P. Y. Arnould

**Affiliations:** ^1^ School of Life and Environmental Sciences, Deakin University, 221 Burwood Hwy, Burwood, VIC 3125, Australia; ^2^ Centre d'Etudes Biologiques de Chizé (CEBC), UMR 7372, CNRS, La Rochelle Université, 79360 Villiers-en-Bois, France; ^3^ Littoral Environnement et Sociétés (LIENSs), UMR 7266, La Rochelle Université, 2 Rue Olympe de Gouges, 17000 La Rochelle, France; ^4^ Institut Universitaire de France (IUF), 1 Rue Descartes, 75005 Paris, France

**Keywords:** distribution, foraging behaviour, tracking, stable isotopes, migration, *Pachyptila turtur*

## Abstract

Effective conservation assessments require detailed information of species' ecological niches during the whole annual cycle. For seabirds, this implies investigating the at-sea distribution and foraging behaviour during both the breeding and non-breeding periods. However, until recently, collecting information about small species has been precluded by the excessive size of the required devices. This lack of knowledge is exacerbated in the case of polytypic genera with species sharing very similar appearance and behaviour, such as the super-abundant prions (*Pachyptila* spp.). The present study investigates the year-round at-sea distribution and foraging ecology of the fairy prion (*Pachyptila turtur*) in southeastern Australia. Miniaturized GPS loggers during the breeding season and geolocators (GLS) during the non-breeding period were used over 4 consecutive years (2017–2021), with results that highlight the importance of the continental shelf-edge waters for fairy prions throughout the year. In addition, contrary to previous assumptions, the GLS data revealed an unsuspected post-breeding migration to the waters south of Australia, during which individuals probably undergo a rapid moult of flight feathers. Understanding the at-sea distribution and ecology of prions during the whole annual cycle will be fundamental to their conservation as it can reveal species- or population-specific threats that have been overlooked because of their status as abundant species.

## 1. Introduction

Seabirds often forage in a patchy and dynamic environment, occupying a wide range of ecological niches [1,2]. Among them, the Procellariiformes are one of the most endangered avian groups [3] owing to the numerous natural and anthropogenic threats faced throughout their distribution [4]. Effective conservation requires precise information of species’ ecological niches during the whole annual cycle, covering both the breeding and non-breeding seasons [5]. However, while studies on large and easily accessible species (albatrosses, large petrels and shearwaters) have benefited from the development of animal-borne data loggers for more than three decades [6], knowledge of the ecology of smaller species has remained limited [7]. The absence of information on their at-sea distribution or foraging ecology has greatly impacted our ability to delineate proper conservation planning [8].

This knowledge bias is exemplified by the super-abundant prions (*Pachyptila* spp.), a group of six to seven small (less than 150 g) pelagic seabird species. Despite being the most abundant seabirds in the Southern Hemisphere (90 million individuals; [9,10]), information on their at-sea distribution is very limited. This is exacerbated by the difficulty of visually differentiating prion species at sea due to the similarity in appearance and behaviour [11]. More recently, advances in the production of small light-level geolocators (GLS) have enabled the study of some of the largest prion species [12–17]. Although this technique provides valuable information about the year-round distribution of seabirds [18,19], the low spatial resolution and temporal frequency of GLS data make it less appropriate to investigate at-sea movements during the breeding season [20]. The lack of precise knowledge regarding the at-sea distribution during the whole annual cycle is, therefore, a matter of conservation concern, especially for such ubiquitous polytypic species.

The fairy prion (*Pachyptila turtur*) is the smallest (110–130 g) of the *Pachyptila* genus [9]. The majority of the species' population breeds in New Zealand and southeastern Australia, but fairy prions also occupy several subantarctic islands of the Atlantic and Indian oceans [9]. In southeastern Australia and New Zealand, fairy prions are considered to forage year-round within a few hundred kilometres of their breeding site [21], feeding predominantly on the euphausiid coastal krill (*Nyctiphanes australis*; [22]). This euphausiid species plays a major role in the high abundance of marine wildlife in these regions, being an essential food source for fish [23], seabirds [24] and whales [25]. However, as a key zooplankton species [26], any variability in abundance or distribution is likely to affect the foraging ecology and fitness of marine predators.

Southeastern Australia is among the most rapidly changing oceanic regions, characterized by strengthening currents, increasing storm frequency and warming sea surface temperature [27]. Particularly, the intensification of marine heatwaves (in frequency, duration and magnitude; [28]) greatly disrupts the zooplanktonic communities [29], potentially affecting a wide range of taxa [30]. By bottom-up controls, the important decrease in zooplankton abundance during events of extreme rise of sea temperatures is likely to induce changes in the foraging behaviour of seabirds [31]. In particular, the sensitivity of coastal krill to marine heatwaves [23,32], and the predicted future increase in duration and frequency of such events in response to global change [28], may directly affect species such as fairy prions [33].

In southeastern Australia, Bass Strait hosts nearly 60% of Australian seabirds, of which the great majority rely almost exclusively on coastal krill [22,34]. In this rapidly changing environment, the threat of food shortage exposes fairy prions to an increased risk of intra- and interspecific competition. Therefore, collecting information detailing the at-sea distribution and foraging ecology of this cryptic species is necessary to better understand and identify the fundamentals of fairy prion ecology. The objectives of this study were to describe the year-round at-sea movements of fairy prions in southeastern Australia during: (i) the incubation and chick-rearing periods through GPS deployments and (ii) the non-breeding period using GLS tracking. In addition, these data were combined with stable isotope analyses to investigate the trophic niche of fairy prions during the breeding period (using whole blood) and the non-breeding period (using body feathers). Finally, the moulting patterns of flight and body feathers were explored using GLS and stable isotope information, as these aspects are critical in the annual cycle of seabirds.

## 2. Methods

### 2.1. Study site, animal handling and instrumentation

The study was conducted during the incubation and chick-rearing periods of breeding (October–January) over 4 consecutive years (2017–2021) and the non-breeding period (February–October) over 2 consecutive years (2019–2020) at Kanowna Island (39°15′ S, 146°30′ E) in northern Bass Strait, southeastern Australia. Fairy prions breeding in this location displayed a synchronous onset of each breeding period (within a range of 11 days) over the 4 study years (laying: 14–24 October; hatching: 30 November–10 December and fledging: 16–27 January) [33]. Seven seabird species breed on this island [35], including 1000–4000 breeding pairs of fairy prions, which represent 1–4% of the Bass Strait population [36].

To evaluate the at-sea movements of fairy prions during the breeding season, adult breeding birds (*N* individuals = 14 and *n* trips = 15 in incubation; *N* individuals = 31 and *n* trips = 58 in chick-rearing) were equipped with a miniaturized GPS logger (nanoFix-GEO, Pathtrack Ltd, Otley, UK), attached to two central tail feathers using waterproof tape (Tesa 4651; Beiersdorf AG). Each individual was tracked either during the incubation or the chick-rearing period, but never during both periods within the same breeding season. The GPS loggers were programmed to record locations at 20 and 10 min intervals during the incubation and chick-rearing periods, respectively. The total mass of logger attachments corresponded to 2.4 ± 0.2% of body mass (124 ± 10 g), which was unlikely to have impacted the foraging and breeding parameters of the equipped individuals [31]. Individuals were weighed (±2 g; Pesola), and bill, tarsus (±0.1 mm; Vernier callipers) and wing length (±1 mm; ruler) were measured.

To determine the at-sea distribution of fairy prions during the non-breeding period, adult birds (*N* individuals = 21) were equipped with leg-mounted GLS (Migrate Technology, model C65, UK). The total mass of logger attachments corresponded to 1.2 ± 0.1% of body mass of the equipped birds. Breeding individuals were equipped during the breeding season and were recaptured during the following breeding season (individuals were captured when attending their burrow). In addition, the timing of wing moult was inferred from information provided by the GLS on the daily proportion of time spent on the sea surface (wet and dry sensor; sampled every 30 s and summarized by 4 h blocks). Since flight feather renewal directly affects the flying ability [37], the peak of time spent on the water during the non-breeding period was used to identify the period when fairy prions moult their wing flight feathers. Finally, the dates of last and first burrow attendance, as well as the periods of the burrow attendance during the non-breeding period, were determined by combining information from the GLS of activity (wet–dry) and movement data (the presence of the bird in the breeding area) [38]. When a burrow attendance was detected during the non-breeding period, it was not possible to confirm that the individual had returned to the exact same breeding colony due to the low spatial accuracy of GLS [20]. However, fairy prions are highly philopatric at the colony level [39] and therefore, it was assumed that tracked individuals were attending burrows at their colony during the non-breeding season.

When individuals were recaptured, six body feathers and blood (0.2 ml from the brachial vein) were collected for the stable isotope analysis. Stable isotope ratios of carbon (δ^13^C) and nitrogen (δ^15^N) in whole blood and body feathers were used as proxies of the foraging habitat and diet/trophic level, respectively. Specifically, isotopic values of whole blood (hereafter blood) reflect the dietary integration of approximately two–four weeks, while body feathers reflect dietary intake when they were synthesized [40] from the end of the breeding season and throughout the non-breeding period [9]. Therefore, isotopic values of blood were used to determine the trophic ecology of the fairy prion throughout the breeding season (incubation and chick-rearing), while body feathers were used for the non-breeding period. One whole body feather per individual was analysed to determine the inter-annual variations. In addition, in 2018–2019, four whole body feathers per individual were analysed to investigate the intra-individual variation.

### 2.2. Data processing and analyses

All data analyses were processed within the R statistical environment [41]. For GPS data, a speed filter with a threshold at 20 m s^−1^ was applied to remove erroneous locations [42]. Because of poor satellite reception when the birds are feeding or sitting on the water, linear interpolation was applied to correct for unequal sampling frequencies between the foraging and commuting. For each complete foraging trip (defined as the time spent at sea between the departure from, and the return to, the burrow), the following basic parameters were calculated: trip duration (h), total horizontal distance travelled (km) and maximum distance from the colony (km). Incomplete trips, where birds started to return towards the colony but the device stopped before the end of the trip, were only used to estimate the maximum distance from the colony. During the chick-rearing period, trips were classified in two categories based on the data distribution of the trip duration: short (less than or equal to 2 days at sea) or long (greater than 2 days at sea). A dual foraging strategy (alternating short and long trips) is common in procellariiform species, including prions [43].

The expectation maximization binary clustering (EMBC) was used to infer the at-sea foraging behaviour of the fairy prion (R package *EMbC*; [41,44]). This method classifies four different movement types based on the travel speed and turning angle between subsequent locations: travelling–commuting (high-speed low turn, HL), resting on the water (low-speed low turn, LL), intensive (low-speed high turn, LH) and extensive searching (high-speed high turn, HH). This method has been shown to be well suited to interpreting ecologically meaningful behaviours from movement data for a range of procellariiform species [45–47].

Processing and calculations of GLS data were conducted using the *GeoLight* package in the R statistical environment [41,48]. The device records the maximum light intensity for each 5 min interval, and the determination of morning and evening twilights enables the longitude (timing of local midday and midnight) and latitude (duration of day and night) to be estimated, providing two positions per day with an average accuracy of 186 ± 114 km (mean ± s.d.; [20]). Before spatial analyses were conducted, data for two weeks before and after the autumn and spring equinoxes were excluded because latitude estimations around these periods are unreliable [49]. The dates of last and first burrow attendance were determined by combining movement data (presence of the bird within 200 km from the breeding colony), information on activity (wet–dry: 100% dry for a period greater than 8 h) and/or light sensor (continuous darkness during the day time). These data were then used to estimate the duration and the total distance travelled during the post-breeding migration. The moulting period of flight feathers was determined for each individual using the period of the maximum proportion of time spent on the water (wet–dry sensor being wet greater than 90% per day; [37]). Wet–dry data were sampled every 30 s with the number of samples wet and maximum conductivity recorded every 4 h.

For both GPS and GLS data, filtered locations were used to generate kernel utilization distribution (UD) estimates. For GPS data, a smoothing parameter of *h* = 0.2 was used with a grid of 0.1° × 0.1° cells (to avoid over-fragmentation), while for GLS data, a *h* of 1.8 was selected (corresponding to a search radius of approx. 200 km) with a 1° × 1° grid cell size (based on the mean accuracy of the device). The 50% (core foraging area) and 95% (home range) kernel UD contours were obtained. Spatial analyses were performed using the *adehabitatHR* R package [41,50].

For stable isotope analyses, blood samples were freeze-dried, ground to powder and homogenized. Body feathers were cleaned of surface lipids and contaminants using a 2 : 1 chloroform : methanol solution in a ultrasonic bath, followed by two successive methanol rinses and air dried 24 h at 50°C [51]. Each feather was then cut with scissors to produce a fine powder for homogenization before the carbon and nitrogen isotope ratio determination using a continuous flow mass spectrometer (Delta V Plus or Delta V Advantage both with a Conflo IV interface, Thermo Scientific, Bremen, Germany) coupled to an elemental analyser (Flash 2000 or Flash EA 1112, Thermo Scientific, Milan, Italy) at the LIENSs laboratory (La Rochelle Université, France). Stable isotope values were expressed in conventional notation (δ*X* = [(*R*_sample_/*R*_standard_) − 1]) where *X* is ^13^C or ^15^N and *R* represent the corresponding ratio ^15^N/^14^N or ^13^C/^12^C. *R*_standard_ values were based on Vienna Pee Dee Belemnite for ^13^C and atmospheric nitrogen (N_2_) for ^15^N. Replicates of internal laboratory standards (Caffeine USGS-61 and USGS-62) indicate measurement errors less than 0.10‰ for δ^13^C and 0.15‰ for δ^15^N.

All statistical analyses were conducted in the R statistical environment [41]. Inter-annual variations in trip parameters (trip duration, total distance travelled, maximum distance travelled and migration duration), behaviours (HH, HL, LH and LL) and isotopic values (whole blood and feathers) were tested using analyses of variance, and *post hoc* tests were conducted using *t*-tests (parametric), or Kruskal–Wallis and Mann–Whitney *U*-tests (non-parametric) depending on the data distributions. Spearman's rank correlation was used to test whether there was a correlation between the maximum range and total distance travelled for GPS tracking data (incubation and chick-rearing). To investigate inter-annual variations in the at-sea distribution during the breeding season (incubation, chick-rearing short and long trips), the percentage overlap in the foraging distribution was estimated using Bhattacharyya's affinity (BA) index [52] using the *adehabitatHR* R package [50]. The BA index (0 signifying no overlap in UDs and 1 signifying complete overlap) is a statistical measure for the degree of similarity among UDs, and the amount of space-use shared among years.

## 3. Results

### 3.1. At-sea behaviour during the breeding season

**Figure 1.**
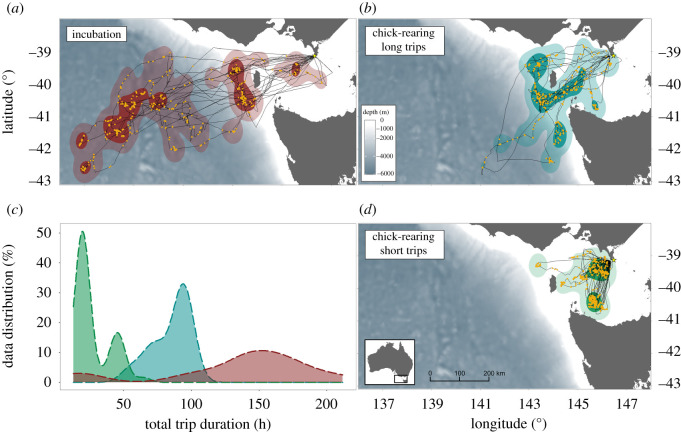
Foraging kernel density distribution is estimated from GPS locations of fairy prions in incubation (*a*) and chick-rearing (*b*,*d*) from Kanowna Island, south eastern Australia; (*c*) shows the distribution density of total trip duration for the incubation (red) and chick-rearing trips (long = blue; short = green). Dark and faded kernel areas show 50% and 95% of the kernel UD, respectively (core area and home range, respectively). Yellow dots indicate when the birds were in intensive foraging (low speed, high turn).

A total of 73 trips (15 during incubation and 58 during chick-rearing) from 45 birds were obtained. During the incubation period, the trip duration was greater than 5 days (max. 9 days, *n* = 13) in all cases, except for two 1-day trips (table 1). These short incubation trips were restricted to 150–200 km from the colony whereas, during the majority of incubation trips, all individuals foraged outside Bass Strait (figure 1), travelling 300–900 km westward to the continental shelf-edge and/or farther west to deep (depth greater than 5000 m) pelagic regions. The trip duration was strongly correlated with maximum range from the colony (Spearman test: *ρ* = 0.87, *p* < 0.001) and total distance travelled (*ρ* = 0.90, *p* < 0.001).

**Table 1.** Trip parameters of the GPS tracking (mean ± s.d.) of fairy prions during the incubation and chick-rearing periods at Kanowna Island, south eastern Australia. The proportions of the different behaviours were determined using the EMBC method. LL, low-speed low turn; LH, low-speed high turn; HL, high-speed low turn; HH, high-speed high turn. See electronic supplementary material, table S1 for inter-annual comparisons.

		incubation	short trips	long trips
(*N* individuals; *n* trips)		*N* = 14; *n* = 15	*N* = 27; *n* = 47	*N* = 11; *n* = 11
trip duration (h)		137 ± 57	27 ± 13	89 ± 10
total distance travelled (km)		1506 ± 715	273 ± 148	1082 ± 306
maximum distance from the colony (km)		545 ± 252	108 ± 55	333 ± 124
behaviours proportion (%)	HH	3.4 ± 2.1	6.5 ± 4.4	9.1 ± 5.1
	HL	47.3 ± 9.0	46.7 ± 12.0	54.6 ± 10.5
	LH	5.3 ± 3.2	13.1 ± 7.9	12.1 ± 6.2
	LL	44.0 ± 11.5	33.7 ± 9.9	24.3 ± 10.7

During the chick-rearing period, individuals alternated between a period of multiple short trips (less than or equal to 2 days) and one long trip (3–4 days) (table 1). Short chick-rearing trips represented the majority of foraging trips (81%) and were all located within Bass Strait (50–200 km from the colony, depth less than 100 m; figure 1). During long foraging trips, individuals mainly foraged along the continental shelf-edge, 250–300 km west and southwest from the colony (figure 1).

**Figure 2.**
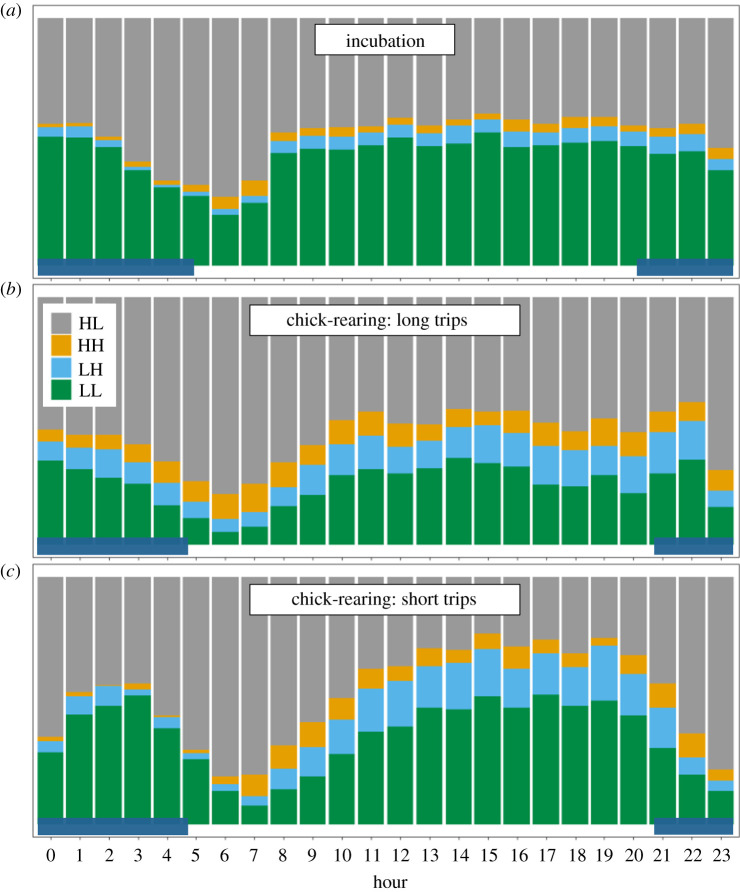
Hourly proportion of the different behaviours of fairy prions during the incubation (*a*) and chick-rearing trips ((*b*) long and (*c*) short trips). LL, low-speed low turn; LH, low-speed high turn; HL, high-speed low turn; HH, high-speed high turn. The dark blue area at the bottom of each panel corresponds to night-time (from after the last lights to before the first lights).

For all foraging trips, during the incubation and chick-rearing periods, trips were characterized by long commuting movements interrupted by the active area-restricted search behaviour. Of the four behavioural categories determined with the EMBC methods, travelling (high-speed low turn; 48 ± 11%) and resting (low-speed low turn; 34 ± 12%) proportionally represented the most dominant behaviours (table 1), while intensive (low-speed high turn; 11 ± 8%) and extensive searching (high-speed high turn; 6 ± 4%) represented a smaller proportion of at-sea behaviours. The proportion of time spent in the intensive foraging was overall higher during the day than the night (figure 2; electronic supplementary material, table S2; *t*-tests: *t*_125.09_ = 4.756, *p* < 0.001). Conversely, individuals spent proportionally more time travelling at night than during the day (*t*-tests: *t*_112.8_ = −5.863, *p* < 0.001). During both incubation and chick-rearing (short and long trips), individuals spent proportionally more time flying around sunrise (figure 2).

**Figure 3.**
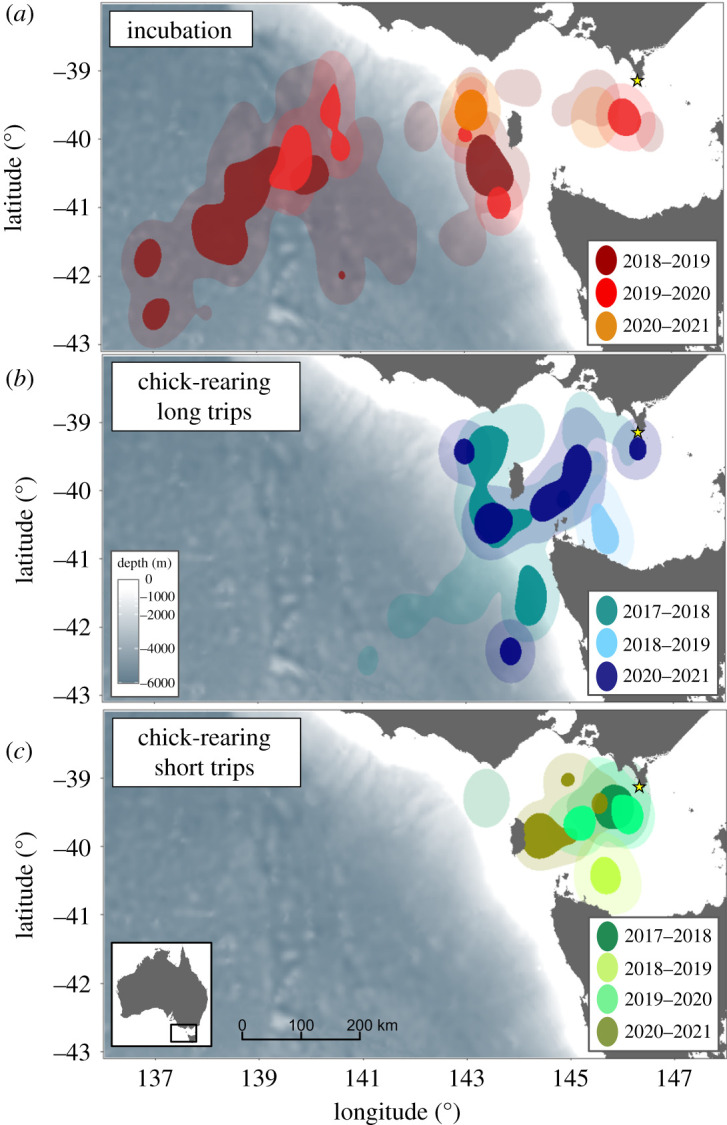
Inter-annual variation of the foraging distribution of fairy prions in incubation (*a*) and chick-rearing ((*b*) long and (*c*) short trips) from Kanowna Island, south eastern Australia. Each panel shows the foraging kernel density distribution estimated from GPS locations. Dark and faded kernel areas correspond to 50% (core area) and 95% (home range) of the kernel UD, respectively.

There was a limited inter-annual variation in the at-sea distribution during the incubation period and the long trips during the chick-rearing period (figure 3). However, for these two groups, the low sample size precluded further statistical comparisons (electronic supplementary material, table S1). For short trips during the chick-rearing period, individuals displayed the important variations between years, both in the at-sea distribution (the BA index less than 0.1) and trip parameters (figure 3; electronic supplementary material, table S1). During the two consecutive breeding seasons of 2018/2019 and 2019/2020, individuals foraged twice as far from the colony than in 2017/2018 and 2020/2021, which was associated with longer trip durations and total distance travelled per trip (electronic supplementary material, table S1). Similarly, individuals spent proportionally more time for travelling and less time for foraging in 2018/2019 and 2019/2020 than in 2017/2018 and 2020/2021 (electronic supplementary material, table S1).

### 3.2. At-sea movements during the non-breeding season

**Figure 4.**
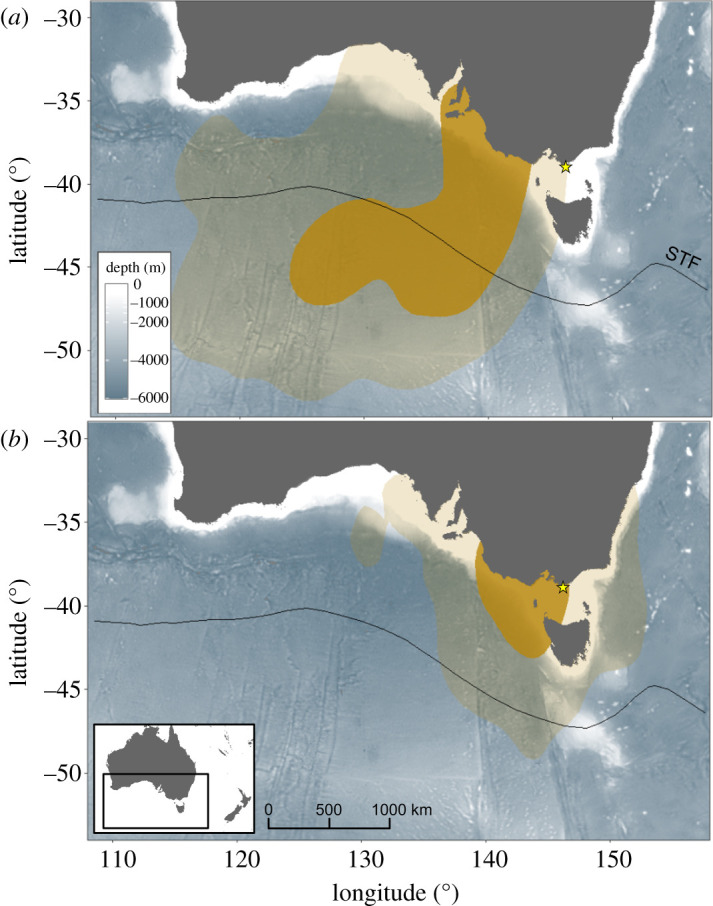
Kernel density distribution is estimated from GLS locations during the non-breeding period of fairy prions from Kanowna Island, south eastern Australia. (*a*) post-breeding migration (from the last burrow attendance to the first burrow attendance; December–June); (*b*) non-breeding period from the first burrow attendance to the start of the following breeding season (February–October). STF, subtropical front [53].

A total of 35 GLS were deployed, of which 23 were retrieved (65.7% retrieval rate). Four GLS were excluded from the spatial analyses as they only recorded for a short period of time after deployment (less than three months). After the end of the breeding period, all the individuals tracked with GLS devices migrated west or southwest of Bass Strait (figure 4). The post-breeding departure ranged from mid-November to early February, depending on the breeding success of the individuals (failed versus successful breeders). All successful breeders (62% of all equipped individuals) departed between 22 January and 3 February. Both failed and successful breeders travelled west 1–2 days after their last burrow attendance and moved rapidly 1500–2500 km from the colony (electronic supplementary material, figure S1). The peak of maximum distance from the colony coincided with a higher daily proportion of time spent on the water (electronic supplementary material, figure S1).

After two–three months spent in the distal area of their post-breeding migration, individuals moved back towards Bass Strait and returned to a burrow on average 125 ± 39 days after their last burrow attendance (table 2; electronic supplementary material, figure S1). Although failed breeders returned slightly earlier than successful breeders (114 ± 49 and 134 ± 31 days, respectively; Mann–Whitney *U*-test: *U* = 67, *p* = 0.153), both groups displayed important inter-individual variation with birds returning to the colony for the first time from late February to early July. Once individuals return to the colony, and for the rest of the non-breeding period, all individuals remained in closer proximity to the colony than during the post-breeding migration. The core distribution area (50% kernel UD) was restricted to the western part of Bass Strait and along the continental shelf-edge (figure 4). During this period, the daily proportion of time spent on the water by individuals decreased to under 50% (electronic supplementary material, figure S1).

**Table 2.** Trip parameters of GLS tracking (mean ± s.d.) of fairy prions during the non-breeding periods at Kanowna Island, south eastern Australia.

	2018–2019	2019–2020
*N* individuals	*N* = 14	*N* = 7
post-breeding migration duration (d)	139 ± 38	94 ± 18
total distance travelled (km)	67 522 ± 10 988	63 164 ± 23 312
maximum distance from colony (km)	1745 ± 334	2010 ± 408

### 3.3. Isotopic niche

**Figure 5.**
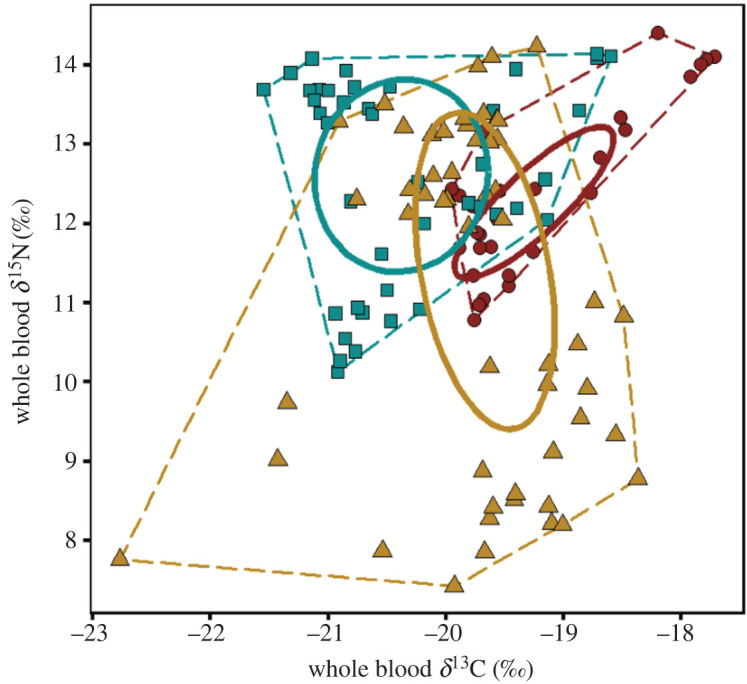
Whole blood and body feather δ^13^C and δ^15^N values of fairy prions from Kanowna Island during the incubation (red dots), chick-rearing (blue squares) and post-breeding periods (yellow triangles). Full lines correspond to the 40% ellipse and dashed lines correspond to the 100% total convex hull. In order to allow statistical comparisons between blood and feathers, isotopic values of feathers were corrected using mean correction factors from Cherel *et al*. [54].

There was a substantial variation in stable isotope values for both blood (incubation and chick-rearing periods) and feathers (non-breeding period) (figure 5). For blood samples, the large isotopic niche occupied by fairy prions was mostly explained by the important inter-annual variation, for both δ^13^C and δ^15^N (table 3). In particular, during chick-rearing, δ^15^N varied from 11.0 ± 0.7‰ in 2018–2019 to 13.5 ± 0.5‰ in 2017–2018 (electronic supplementary material, figure S2). Conversely, stable isotope values in body feathers did not vary significantly between years (table 3). Instead, within each year, the analysis of one body feather per individual resulted in a substantial inter-individual variation for both δ^13^C and δ^15^N. However, the analysis of four feathers per individual in 2018–2019 revealed large intra-individual variations in δ^13^C and δ^15^N, exceeding 1.7 and 6.7‰, respectively (electronic supplementary material, figure S3).

**Table 3.** Whole blood and body feather δ^13^C and δ^15^N values (means ± s.d.) of fairy prions from Kanowna Island, south eastern Australia. Significantly different values (Mann–Whitney *U*-test: *p* < 0.05) are indicated by different superscript letters/symbols, for comparison between years (row; *, # or &), and between incubation and chick-rearing for whole blood values (columns; a or b).

		inter-breeding (feathers)	incubation (blood)	chick-rearing (blood)
δ^13^C (‰)	2017–2018	−18.0 ± 0.8* (*n* = 18)	—	−20.9 ± 0.3* (*n* = 17)
	2018–2019	−18.1 ± 0.9* (*n* = 20)	−19.8 ± 0.1^a*^ (*n* = 10)	−20.6 ± 0.4^b#^ (*n* = 12)
	2019–2020	−17.7 ± 0.6* (*n* = 15)	−18.9 ± 0.8^a#^ (*n* = 18)	−19.3 ± 0.5^a&^ (*n* = 13)
δ^15^N (‰)	2017–2018	12.2 ± 1.9* (*n* = 18)	—	13.5 ± 0.5^a*^ (*n* = 17)
	2018–2019	12.0 ± 2.3* (*n* = 20)	11.9 ± 0.7^a*^ (*n* = 10)	11.0 ± 0.7^b#^ (*n* = 12)
	2019–2020	12.6 ± 2.3* (*n* = 15)	12.6 ± 1.1^a*^ (*n* = 18)	12.9 ± 1.0^a*^ (*n* = 13)

## 4. Discussion

Using 4 years of GPS tracking, the present study provides the first detailed information of at-sea movements during the breeding season of a species from the genus *Pachyptila*, the fairy prion. In addition, while this species was not known to migrate during the non-breeding period [21], the analysis of winter distribution revealed a clear post-breeding migration during the first three–five months of this period. This detailed information, combined with the data on their isotopic niche and moult patterns, provides crucial knowledge about a super-abundant, but still under-studied, genus.

### 4.1. Breeding season

During the breeding season, individuals foraged primarily along the continental shelf-edge 200–300 km from the colony, but also used pelagic waters (500–900 km from the colony) and continental shelf areas (within Bass Strait). This distribution matches at-sea observation of prions during boat surveys [55], with high concentrations of individuals found along the productive waters of northwest Tasmania [56]. This area is part of the Great South Australian Coastal Upwelling System [57], which is a key feature for several seabird species such as the shy albatross (*Thalassarche cauta*; [58]).

During this period, the maximum foraging distance of fairy prions from Kanowna Island appeared to be in the same range as other species of the genus *Pachyptila* elsewhere [12,17]. This range is likely to be correlated with the threshold between finding a profitable foraging area without further extending the duration of the trip [43]. However, fairy prions from Poor Knights Islands (New Zealand) performed substantially shorter trips during incubation compared with those in the present study (2.4 and 5.7 days, respectively; [21]). This corresponds to the presence of a more local foraging area in northern New Zealand compared with Kanowna Island, mirroring the differences in the foraging behaviour observed between the populations of common diving petrels (*Pelecanoides urinatrix*) from these two breeding areas [31,59].

During the chick-rearing period, individuals repeated short trips of 1–2 days and long trips of 3–5 days. Despite some inter-annual variations, short trips were exclusively performed within Bass Strait, west and southwest of the colony. During long trips, individuals travelled farther west, along the continental shelf-edge. Most procellariiform species use a similar bimodal foraging strategy when rearing chicks, alternating short trips close to the colony with longer foraging trips to greater distances [43]. Such a strategy appears to be a trade-off between provisioning chicks regularly and maintaining the adult body condition throughout the breeding season. Net energy transfer to the chicks is higher with short trips but at the cost of adult's body reserves [43]. During long trips, adults restore their body condition by foraging in or towards highly productive areas such as frontal zones or shelf slopes [60,61].

During both incubation and chick-rearing trips, individuals spent a greater proportion of time travelling around sunrise. Leaving the nest before the first light is characteristic of small burrowing petrels and is thought to be a strategy to minimize the risk of predation on land [21]. Although the proportion of intensive searching was higher during the day, this activity also persisted at night. Prions are visual predators [12] but, as surface feeders, they rely on the presence of their prey near the surface to access it. At night, despite limited visibility, the vertical migration of euphausiids may make them more available to prions [62]. Similar activity patterns have been observed for Antarctic prions (*Pachyptila desolata*) and blue petrels (*Halobaena caerulea*) [12], for which the increased feeding activity at night was related to their high visual acuity [63].

Results of the stable isotope analysis in blood were characterized by substantial inter-annual variations, especially during the chick-rearing period (present study; [22]). Although such variations may reflect different foraging areas between years [64], this could also suggest modifications in prey size or prey species consumed [65]. During the breeding periods 2018–2019 and 2019–2020, fairy prions exhibited significantly lower δ^15^N blood values than in 2017–2018, which also corresponded to longer foraging trip durations. A similar pattern was observed with the sympatric common diving petrel, which was explained by the cascading effects of marine heatwaves in southeastern Australia [31]. During these two successive breeding seasons, the warmer sea surface temperature is likely to have disrupted the availability of their main prey, the coastal krill, significantly impacting the breeding success of both prion and diving petrel species [33]. The abnormally warm waters induced a shift of dominant zooplanktonic species from energetically rich large-bodied cold-water euphausiids (such as coastal krill) to lower-quality smaller-size subtropical copepods [29].

However, in contrast with the sympatric common diving petrels, fairy prions appeared to buffer the variations in environmental conditions better, owing to their higher flight capacity and the production of stomach oil [33,66]. Indeed, despite being smaller than common diving petrels, the lower wing load of fairy prions enables them to reach more distant foraging areas [12]. In addition, the production of stomach oil mediates the mass loss of the chick in between meals [67], allowing the adults to extend their foraging trips without detrimental effects to chick survival.

### 4.2. Migration and wintering distribution

After the breeding period, all the tracked fairy prions from Kanowna Island performed a clear migration 1500–3000 km west/southwest of the breeding colony in the vicinity of the subtropical front. Similar to broad-billed (*Pachyptila vittata*), MacGillivray's (*Pachyptila macgillivrayi*) and thin-billed (*Pachyptila belcheri*) prions [14,17], this migration was characterized by a rapid outward movement followed by a relatively stationary period of two–three months at the distal point of the migration before a rapid return movement towards the colony, three–five months after departure. Previously, in contrast with all the other *Pachyptila* species, the fairy prion was considered to not migrate during the non-breeding period [9]. However, this assumption was based only on at-sea observations [21] which did not take into account the breeding status of the birds. In particular, as observed in the present study, adults that failed early during the breeding season returned from migration only a few weeks after the last successful breeders left the colony. This may result in a perceived continuous presence of the species at the colony and in the surrounding waters. These findings emphasize the importance of tracking data in understanding the distribution of seabird species, especially during the critical non-breeding period.

Soon after departure, all tracked prions exhibited a marked increase in time spent on the water coinciding with the distal point of their migration. As previously observed in other prion and small petrel species, this reduced flight activity is likely to be related to intense flight feather moult [17,37,38]. This is consistent with the observation at the colony, during the winter period, of birds with fully moulted flight feathers [9]. The occurrence of a flight feather moult period within a few months after departure for all individuals, independent of their breeding output (failed or successful breeders), suggests that this moult may be triggered by the end of individual's reproductive duties. This is similar to the broad-billed prion [17] or the sympatric common diving petrel [38] that shows spatial and inter-annual variation in moult period related to the time of departure in migration.

Body feathers, however, appeared to be renewed throughout a longer period, probably including the late breeding period and most of the non-breeding period. Indeed, results of stable isotope analyses in feathers indicated substantial intra-individual variations. Large differences among body feathers of the same bird suggest that the individual was in different areas when they were synthesized [64]. In the present study, isotopic signatures ranged from coastal to oceanic environments, which is consistent with their migration pattern and the observation of adults starting to moult body feathers before the end of the breeding season and throughout the winter [9]. Thin-billed and Antarctic prions display a similar protracted moult of their body feathers throughout several months [37,68]. A continuous moult of the body feathers allows individuals to progressively renew their plumage while maintaining key aspects of waterproofness and thermoregulation [69].

After returning from migration (in late February–June), individuals stayed within the general area of the colony (less than 1000 km) until the start of the next breeding season. During this period, the at-sea distribution was mostly restricted to western Bass Strait and along the continental shelf-edge, matching at-sea survey observations [55]. The large upwelling systems in this area [70] are likely to contribute to the high availability of coastal krill throughout this period [71]. In autumn, this region is known to host large populations of marine predators feeding predominantly on coastal krill, such as short-tailed shearwaters (*Ardenna tenuirostris*; [72]) and blue whales (*Balaenoptera musculus*; [25]).

During this period and throughout winter, individuals returned to a burrow several times for a duration of one night to 3 consecutive days. Like other prion species, this behaviour could be considered as pre-breeding attendance [73]. However, in the present study, such a scenario seems unlikely as this behaviour occurred up to seven months before the start of the next breeding season. Interestingly, the common diving petrel breeding in the same colony exhibits a very similar pattern, coming back to the colony after a short migration to Antarctic waters and attending a burrow several times throughout the non-breeding period [38]. When present at the colony during the non-breeding period, both species exhibit regular high rates of vocalization at night (Fromant *et al*. 2020, unpublished data), which may be linked to territorial behaviour [74]. The potential inter- and/or intra-specific competition for nesting habitat on this island could induce a greater amount of time being spent in the colony during the non-breeding period. Indeed, in southeastern Australia and New Zealand, fairy prions and common diving petrels are potentially direct rivals for nesting habitat and have been regularly observed fighting in burrows [74].

## 5. Conclusion

The present study highlighted important aspects of fairy prion at-sea distribution and foraging ecology. In particular, the deployment of light sensor GLS revealed an unsuspected post-breeding migration to the south of Australia, during which individuals probably undergo a rapid moult of flight feathers. In addition to this critical post-breeding migration, the tracking data of fairy prions from Kanowna Island highlighted the importance of the continental shelf-edge waters throughout the year, during both the breeding and non-breeding periods. Topographically driven upwellings are stable features resulting in a spatially and temporally reliable food source, and the several upwelling systems occurring off southeastern Australia [70] are essential to support the large avian biomass in this area [58]. However, the recent successive marine heatwaves in the region induced substantial effects on the phenology and breeding success of fairy prions in the region [33], and it is uncertain how upwelling mechanisms and marine heatwaves are related. This is particularly concerning as the frequency and intensity of such extreme events are predicted to increase in the near future [28]. In addition, although recent studies showed that fairy prions are able to maintain a relatively good breeding success even during poor years [33,66], the long-term consequences of marine heatwaves on juveniles' and adults’ survival remain totally unknown.

## Data Availability

Our data are available within the Dryad Digital Repository: https://doi.org/10.5061/dryad.gmsbcc2q6 [75]. Additional material is provided in the electronic supplementary material [76].
